# Effect of nanosilver fluoride versus silver diamine fluoride on shear bond strength and color change of resin-modified glass ionomer cement bonded to artificial dentin caries: an in-vitro study

**DOI:** 10.1186/s12903-026-08294-7

**Published:** 2026-04-23

**Authors:** Mennatullah Khaled Mubarak, Gehan Gaber Allam, Nada Atef Aboushady

**Affiliations:** Department of Pediatric Dentistry and Dental Public Health, Faculty of Dentistry, Ain Shams University, African unity St., Cairo, 11566 Egypt

**Keywords:** Nanosilver fluoride, Silver Diamine Fluoride, Resin-modified glass-ionomer cement, Shear bond strength, Color, Dental caries

## Abstract

**Background:**

Nanosilver fluoride (NSF) has emerged as a promising alternative to silver diamine fluoride (SDF), addressing its major drawback of tooth discoloration. Incorporating silver nanoparticles, NSF retains the advantageous preventive, antimicrobial and remineralizing characteristics of SDF without causing staining. This study aimed to investigate the effect of nanosilver fluoride versus silver diamine fluoride pretreatment on shear bond strength and color of resin-modified glass-ionomer cement (RMGIC) to caries-affected dentin and the impact of thermocycling on shear bond strength.

**Methods:**

Forty-eight primary molars with artificially demineralized dentin were assigned to three groups: Group A (control, bonded with RMGIC), Group B (SDF treatment before RMGIC), and Group C (NSF treatment before RMGIC). Each group was subdivided into two subgroups 8 samples each; in subgroup 1 (non-aged) specimens underwent shear bond strength testing, in subgroup 2 (aged) baseline shade following RMGIC restoration was recorded, then specimens were subjected to 5000 cycles thermocycling prior to shade re-evaluation, color change assessment, and shear testing. Continuous data were presented as mean and standard deviation values. They were examined for normality and variance homogeneity by Shapiro-Wilk and Levene tests. Color change was tested with one-way ANOVA and Tukey’s post hoc, and bond strength with two-way ANOVA. The significance level was set at *p* < 0.05 for all tests.

**Results:**

In non-aged subgroups, NSF demonstrated the highest bond strength, that was significantly higher than the control (*p* < 0.001), but nonsignificant when compared with SDF group; SDF exhibited insignificantly higher values as opposed to control. In aged subgroups, SDF had insignificantly higher values than NSF, and both were significantly higher than control (*p* < 0.001). Non-aged samples showed significantly higher bond strengths to aged samples across all groups. NSF group showed the lowest value of color change (ΔE_00_) (3.28 ± 0.75), followed by control with an insignificant difference (3.34 ± 1.09), SDF exhibited the highest value with a significant difference to other groups (5.03 ± 1.03).

**Conclusion:**

NSF pretreatment resulted in shear bond strength of RMGI to caries-affected dentin comparable to SDF, while yielding significantly less color change after thermocycling. These results suggest NSF as a potential alternative to SDF, highlighting the need for further clinical validation.

## Background

The widespread occurrence of dental caries among children makes it a major global health concern [1, 2]. Dental caries is a complex and dynamic disease that results from the interaction of several contributing factors, notably the dental substrate, the microbial biofilm adherent to the tooth surface and the intake of fermentable carbohydrates. The initiation and progression of the disease are governed by the equilibrium between detrimental and protective influences [3].

Contemporary understanding of dental caries identifies it as a plaque-driven metabolic process that causes tooth demineralization. This has shifted clinical practice from extensive cavity preparation to minimally invasive techniques focused on preserving healthy tissue [4, 5]. In parallel, modern caries management increasingly advocates for the retention of demineralized tissues with the potential for remineralization, alongside non-invasive interventions that minimize or even eliminate the need for tissue removal, thereby aligning with the principles of conservative and preventive dentistry [6].

Among the various approaches encompassed within minimally invasive dentistry (MID), silver diamine fluoride (SDF) has gained substantial prominence [7]. Its pronounced antibacterial activity against cariogenic microorganisms has been well established and systematic reviews confirm its effectiveness in arresting carious lesions, particularly in the primary dentition [1]. Its efficacy is attributed to its potent bactericidal activity, its ability to inhibit demineralization and its capacity to enhance the remineralization of affected dentin. These combined properties not only account for its effectiveness as a non-invasive treatment but also support its potential role as an adjunct in restorative dentistry to minimize the risk of recurrent carious lesions [8]. Despite these advantages, a principal drawback of SDF remains the irreversible black staining of affected dentin and enamel [1].

The silver-modified atraumatic restorative technique (SMART) combines SDF application with restorative intervention for cavitated carious lesions. This technique involves selective removal of softened dentin, followed by restoration with glass-ionomer cement (GIC) [9]. To further enhance the durability and clinical success of this approach, resin-modified glass-ionomer cement (RMGIC) was subsequently introduced, offering superior mechanical performance, enhanced adhesion and reduced sensitivity to moisture compared with conventional GIC [4, 10].

Nanotechnology, an evolving direction in dentistry, has shown promise in advancing anti-caries strategies by incorporating nanoparticles into therapeutic formulation. Their high surface area relative to volume contributes to enhanced biomedical activity. Silver nanoparticles (AgNPs) were of particular importance as they exhibited strong antimicrobial activity against a broad spectrum of microorganisms [11].

Nanosilver Fluoride (NSF), which combines AgNPs, chitosan, and fluoride, has demonstrated both preventive and antimicrobial properties without causing the black staining commonly associated with SDF [2]. NSF is further characterized as a safe, eco-friendly and cost-effective material. The antibacterial mechanism of AgNPs is attributed to their high surface area, which facilitates penetration through bacterial cell walls, leading to disruption of the cell membrane and interference with DNA replication, ultimately resulting in bacterial cell death. Evidence also supports the anti-caries potential of NSF, showing superior efficacy compared to control group in the arrest of early childhood caries [12].

Despite the promising potential of NSF in arresting caries in primary teeth with efficacy comparable to and even surpassing SDF [1, 12], evidence remains limited regarding its influence on the bond strength and color stability of resin-modified glass-ionomer (RMGI) restorations bonded to caries-affected dentin. On the other hand, a recent systematic review showed that SDF pretreatment had no significant effect on the bonding performance of glass-ionomer cement to dentin [8]. Accordingly, this study aimed to evaluate whether NSF could serve as a viable esthetically-appealing alternative to the widely accepted SDF and whether it would overcome the darkening drawback of SDF without interfering with the bonding of RMGIC to caries-affected dentin. The present study focuses on the overall optical outcome of RMGIC and the underlying pretreated dentin with NSF or SDF, given that the final esthetic appearance represents a primary concern for patients rather than the color of dentin substrate alone.

The null hypothesis reports that (1) There is no significant difference in the shear bond strength of RMGIC to artificially induced carious dentin pretreated with either NSF or SDF (2). There is no significant difference in overall color change after thermocycling of RMGIC placed over NSF or SDF-treated dentin (3). There is no significant difference in shear bond strength of RMGIC before and after thermocycling.

## Methods

### Sample size calculation

 A power analysis was conducted to ensure sufficient statistical power for testing the null hypothesis of no difference among the groups with respect to shear bond strength. Using a significance level (α) of 0.05, a beta (β) of 0.1 (corresponding to 90% power), and an effect size (f) of 0.626 derived from a previous study [13], the total sample size required was determined to be 48 specimens. This corresponded to 16 specimens per group and 8 specimens per subgroup. The calculation was performed using R statistical software, version 4.3.2 for Windows [14].

### Ethical considerations

This investigation was exempted from full ethical review by the Research Ethics Committee of the Faculty of Dentistry, Ain Shams University (FDASU-REC), as it was an in vitro study utilizing teeth obtained from anonymous donors. Ethical exemption was granted under reference number FDASU-REC EM122311.

### Sample collection and storage

Seventy extracted primary molars were initially collected under a protocol approved by the Ethics Committee of the Faculty of Dentistry, Ain Shams University, at the outpatient clinic of the Department of Pediatric Dentistry and Dental Public Health. The teeth were obtained from anonymous pediatric patients and were extracted either due to over-retention or for orthodontic purposes. Only forty-eight teeth fulfilling the inclusion criteria, sound dentin or minimal caries confined to the outer enamel without cracks or structural defects, were selected for the study. All specimens were cleaned using a hand scaler, followed by polishing with a rubber cup attached to a low-speed contra-angle handpiece. The teeth were then stored in 0.1% thymol solution at room temperature, with the solution replenished weekly.

### Sample grouping and specimen preparation

**Fig. 1:**
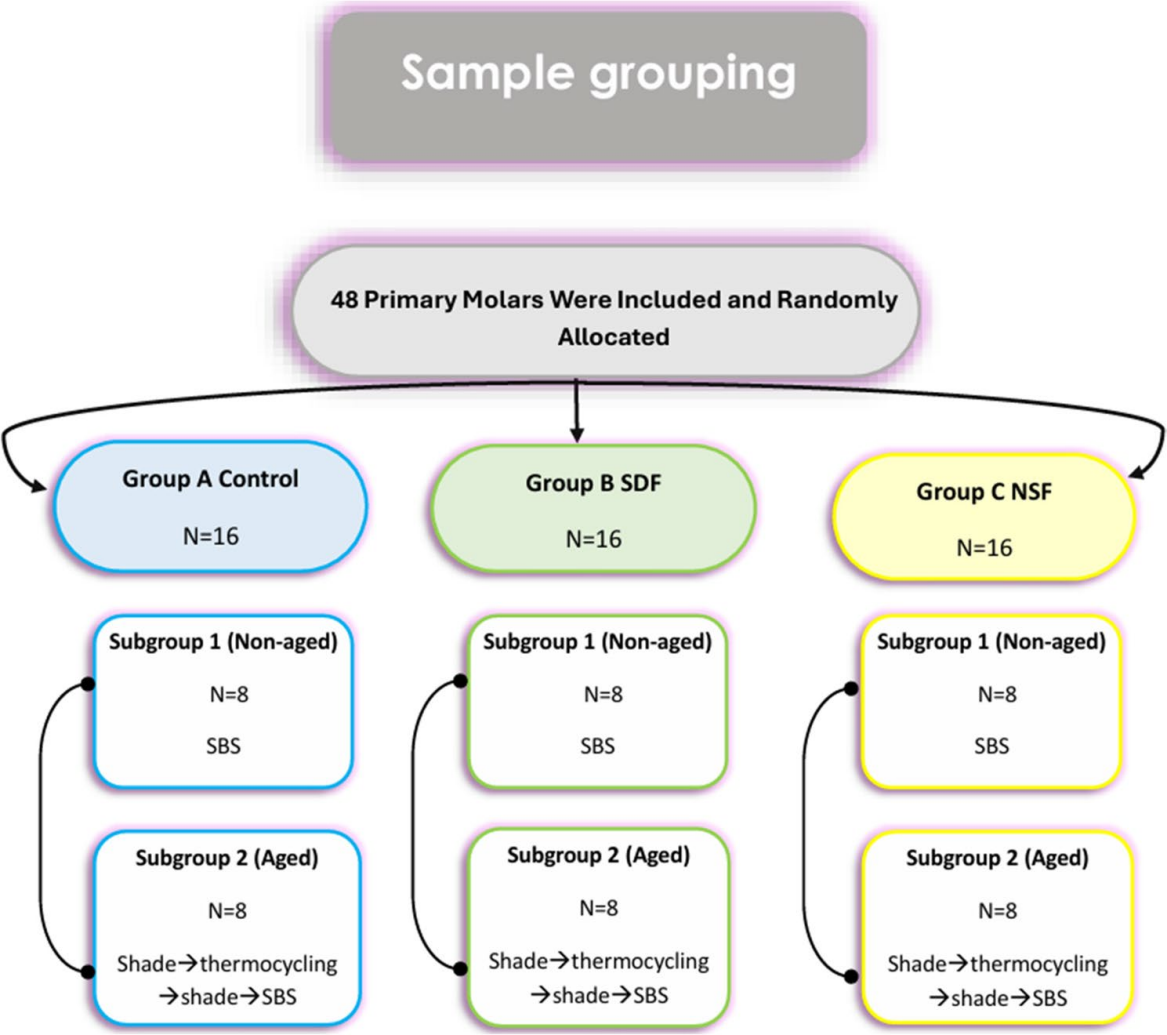
Flowchart of sample grouping

The forty-eight selected specimens were sequentially numbered and randomly allocated into three main groups (*n* = 16 per group): Group A (Control), group B (SDF) and group C (NSF). Each group was further divided into two subgroups (*n* = 8 per subgroup). The specimens were sectioned with a low-speed diamond saw (ISOMET 4000; Buehler Ltd, Lake Bluff, IL, USA) to obtain flat dentin surfaces. The smear layer on the prepared surfaces was standardized by sequential wet polishing with silicon carbide abrasive papers, then each specimen was embedded in self-cured acrylic resin within polyvinyl rings. A 5 mm circular window was created on the occlusal surface by covering the area with adhesive tape while the surrounding surfaces were coated with acid-resistant matt-black nail polish to prevent any untoward interactions. After drying, the tape was removed and specimens were individually numbered. In subgroup 1 (non-aged), specimens were bonded with resin-modified glass-ionomer cement (RMGIC) and subsequently subjected to shear bond strength testing (SBS). In subgroup 2 (aged), specimens were bonded with RMGIC, and their baseline shade was recorded after setting of RMGIC using a Vita Easyshade spectrophotometer. Thereafter, they underwent thermocycling aging for 5000 cycles between 5 °C and 55 °C in controlled water bath with a dwell time of 30 s and transfer time of 15 s in a thermocycling apparatus (SD-Mechatronik, Westerham, Germany), followed by shade re-evaluation and overall color change of RMGIC-dentin complex was assessed then shear bond testing. The experimental design is summarized in Fig. 1.

### Preparation and characterization of Nanosilver Fluoride (NSF)

NSF was synthesized at Nano Gate lab, Cairo, Egypt, through the chemical reduction of silver nitrate (AgNO₃) using sodium borohydride (NaBH₄) with chitosan serving as a stabilizing biopolymer, following the method of Targino et al. [15]. Specifically, 1 mL of AgNO₃ (0.11 M) was mixed with 28.7 mL of chitosan solution (2.5 mg/mL in 1% acetic acid) under continuous magnetic stirring until homogenous. The mixture was cooled in an ice bath and freshly prepared NaBH₄ (0.3 mL, 0.8 M) was added dropwise with vigorous stirring. After removal from the cold bath, sodium fluoride (10,147 ppm) was incorporated to enhance stability and cariostatic properties. The solution was stirred overnight to ensure complete synthesis. Stabilized and sonicated samples were analyzed using transmission electron microscopy (TEM) on a JEOL JEM-2100 (200 kV) [2]. Optical characterization of silver nanoparticles was performed using UV-Vis spectrophotometry [12].

### Artificial caries formation

**Fig. 2:**
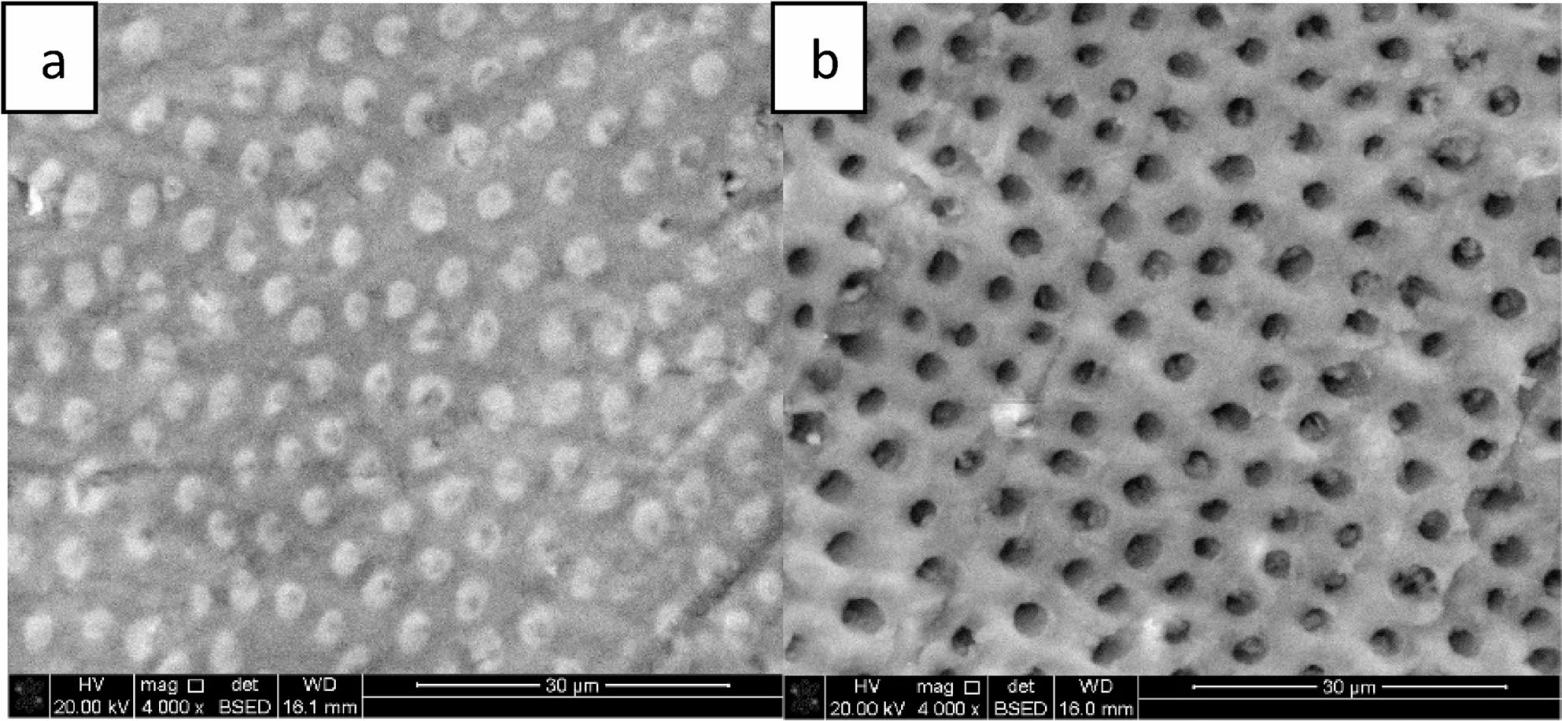
SEM **a**- before artificial demineralization **b**- after artificial demineralization

**Fig. 3:**
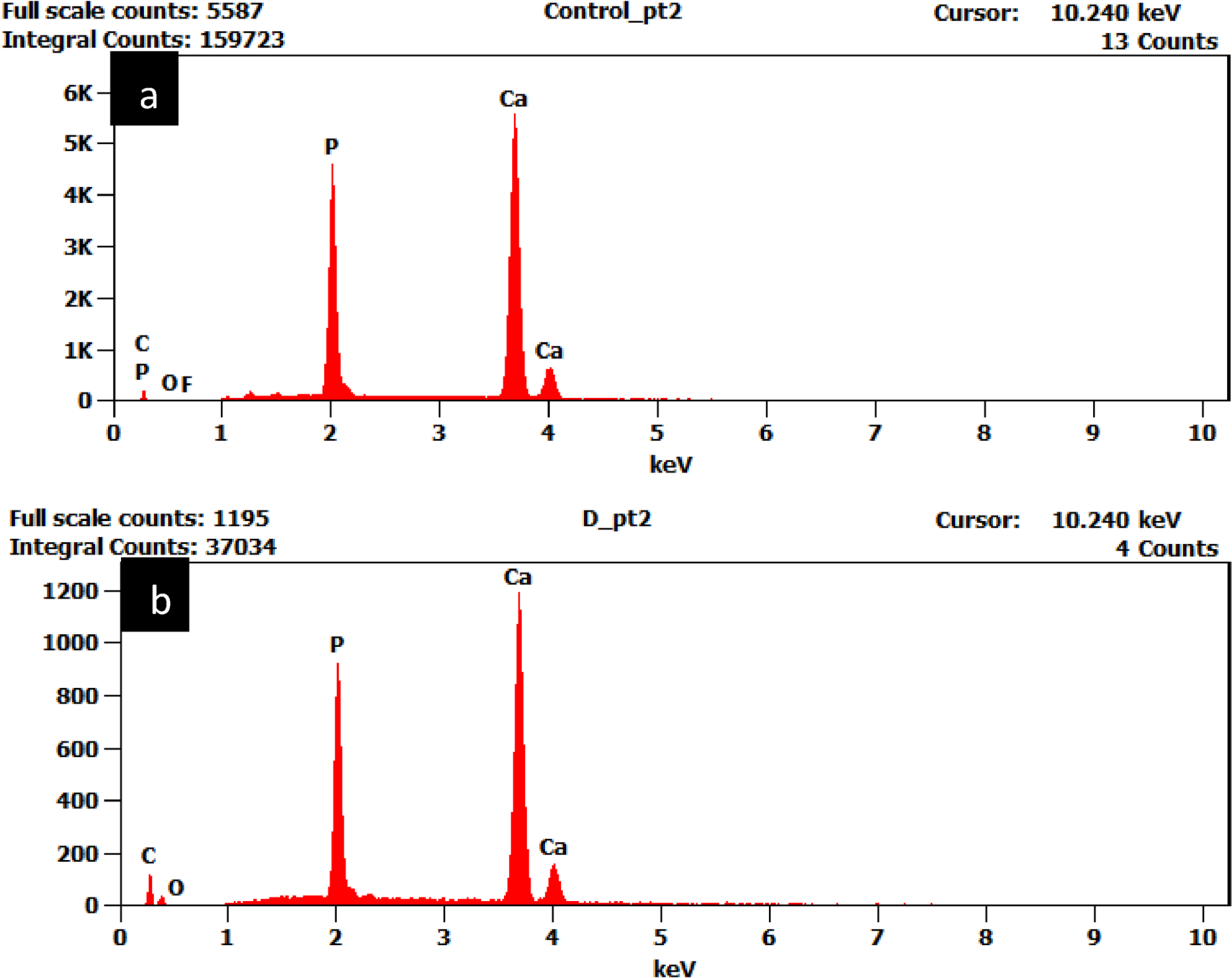
EDX **a**- before artificial demineralization **b**- after artificial demineralization

**Fig. 4:**
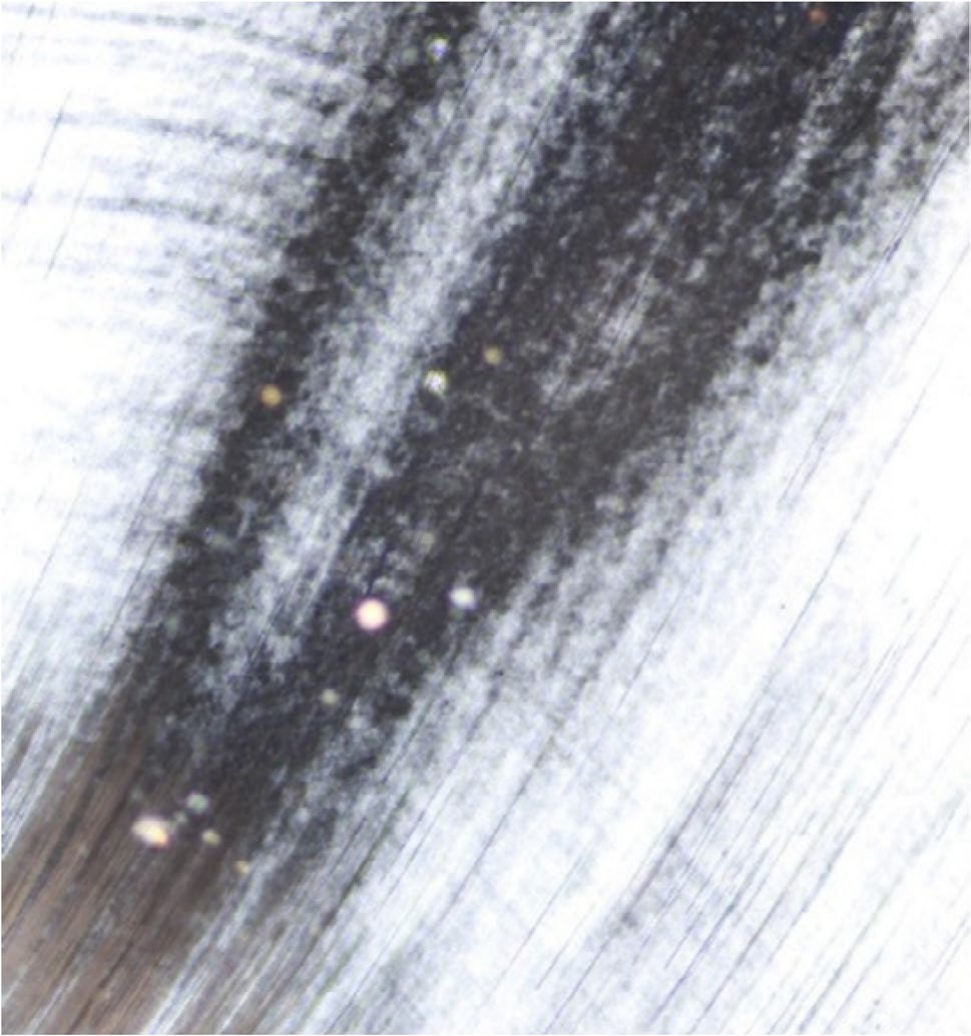
Polarized light microscope showing lesion formation

For artificial caries induction, specimens from all groups were immersed in the demineralizing solution (2 mL/mm²) at room temperature for 96 h and the solution was replaced every 24 h, after which they were rinsed with deionized water. The demineralizing solution (2.2 mM CaCl₂, 2.2 mM KH₂PO₄, 50 mM acetate, pH 4.4) was stored in dark glass containers to prevent light-induced reactions [16]. Its effectiveness was verified in a pilot study by examining teeth using scanning electron microscopy and energy dispersive X-ray spectroscopy (SEM-EDX), where SEM provided morphological assessment and EDX analyzed elemental composition of demineralized dentin surface [17], (Figs. 2 and 3) followed by sectioning and polarized light microscopy assessment (PLM) to confirm lesion formation [18] (Fig. 4).

### Restorative procedures

**Fig. 5:**
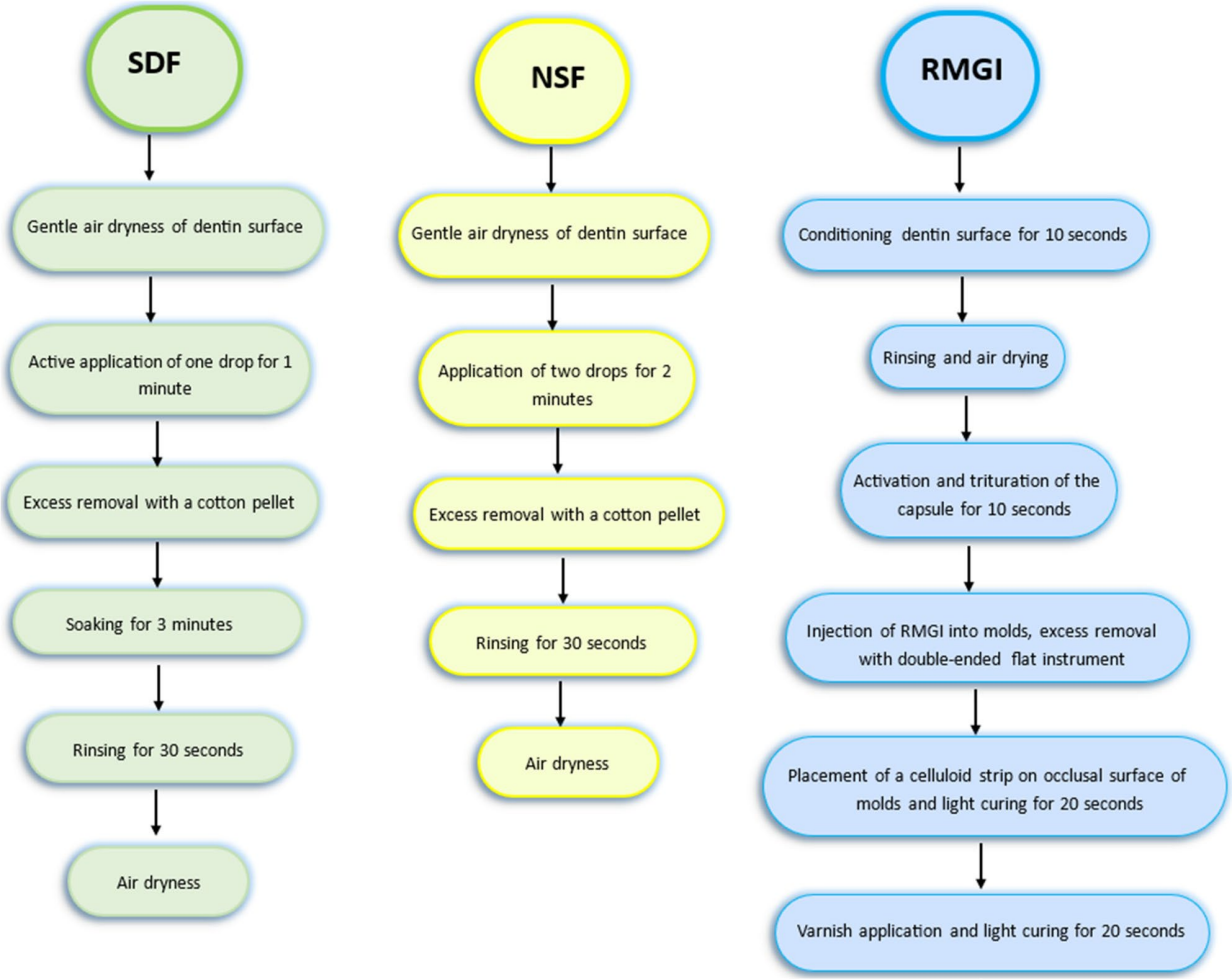
Flowchart of application techniques

The materials used in this study were applied according to the manufacturer’s instruction, their composition and application technique are summarized in Table 1; Fig. 5. All the specimens of group A (Control) were treated first with a conditioner followed by rinsing and air dryness, then a rubber catheter with an internal diameter of 5 mm was placed perpendicular to the dentin surface to serve as a mold for RMGI. Eventually after setting, the rubber catheter was removed and the light cured restoration was painted with nano-filled coating. Regarding the groups B and C, namely SDF and NSF groups, the demineralized dentin surfaces of the former were treated with SDF while specimens of the latter received NSF treatment. Subsequently, the dentin conditioning and restorative procedure was executed as mentioned with the control group.

**Table 1:** Materials, composition and application technique

Material	Composition	Application
*Ketac™ Conditioner*	25% polyacrylic acid (pH = 1.5-2)	Application of a thin layer for 10 s, rinsed with water and gently air dried until a matte glossy surface was achieved.
*e-SDF* ^*®*^	25% silver, 62% water, 5% fluoride, 8% amine (pH = 10)	Active application of one drop for 1 min using a microbrush, excess removal using a cotton pellet followed by rinsing using air-water syringe for 30 s after 3 min interval.
*Nanosilver fluoride*	Chitosan (28.7 ml, 2.5 mg/ml), sodium fluoride (10,147ppm), Ag+ (400ppm)	Application of two drops with a microbrush for 2 min, excess removal with a cotton pellet. Rinsing for 30 s.
*GC Fuji II LC* ^*®*^ *(Resin-modified glass-ionomer cement)*	Powder: 100% fluoro-alumino- silicate Liquid: 35% HEMA, 25% distilled water, 24% polyacrylic acid, 6% tartaric acid and 0.10% camphorquinone	The capsule was activated, triturated for 10 s and loaded into an applicator gun, then the material was injected into molds, excess removed using double-ended flat instrument. A celluloid strip was placed on occlusal surface of molds against which the RMGI was light-cured for 20 s.
*Equia coat*	Methyl methacrylate, colloidal silica, camphorquinone, urethane methacrylate, phosphoric ester monomer	Application of a thin layer then light curing for 20 s.

### Testing procedures

#### A- Macro-shear bond strength testing

Shear bond strength (SBS) was evaluated using a universal testing machine, where a knife-edged chisel applied a shear load at 1 mm/min parallel to the bonded interface of RMGIC buttons, positioned at the minimum achievable distance without direct contact, until debonding occurred. The experiments were performed under dry conditions. The maximum load was recorded in Newtons and converted to megapascals through dividing it by the bonded surface area. Regarding the specimens of subgroup 1 in the three groups (Control, SDF and NSF), shear strength was evaluated following 24-hour storage in artificial saliva to allow complete setting of RMGI. For subgroup 2, testing was conducted after thermocycling and color assessment.

#### B- Color change assessment

Color assessment of subgroup 2 specimens from groups (A, B, and C) was performed using a VITA Easyshade® Advance 5.0 spectrophotometer to record CIELab values at two time points: 24 h post-restoration (T0) and after 5000 thermocycles (T1). Measurements were conducted under standardized conditions with proper calibration, three consecutive readings per specimen and averaged values recorded. The ΔE₀₀ color difference (T1- T0) was calculated using the following CIEDE2000 formula. The terms ΔL′, ΔC′, and ΔH′ represent the differences in lightness, chroma, and hue, respectively. The parametric factors K_L_, K_C_, and K_H_ are used to adjust the calculation according to the different viewing conditions. Additionally, S_L_, S_C_, and S_H_ are the weighting functions that account for the color difference regarding the variation in the location of L*, a*, and b* color coordinates, while RT refers to the rotation function accounting for the interaction between chroma and hue differences, particularly in the blue region of the color space.

$$\triangle E_{oo}=[ (\frac {\triangle L'}{K_L S_L})^2 + (\frac {\triangle C'}{K_C S_C})^2 + (\frac {\triangle H'}{K_H S_H})^2+R_T(\frac {\triangle C'}{K_C S_C})(\frac {\triangle H'}{K_H S_H}) ]^{1/2}$$

### Statistical analysis

Continuous variables were reported as means and standard deviations (SD) and tested for normality and homogeneity of variance using the Shapiro–Wilk and Levene tests, respectively, with all assumptions confirmed. Color change was assessed using one-way ANOVA followed by Tukey’s post hoc test, while bond strength was evaluated with a two-way ANOVA. Simple effects comparisons of estimated marginal means were further examined using the Wald test, applying the model’s error term and Sidak’s adjustment for multiple comparisons.

## Results

### Nanosilver fluoride characterization

**Fig. 6:**
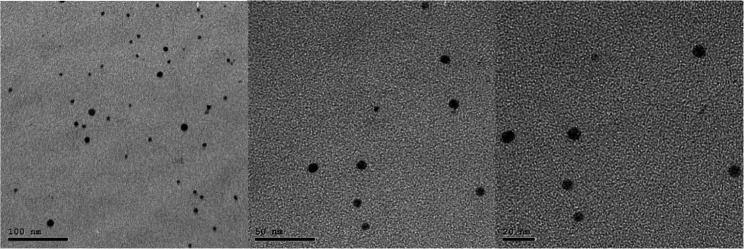
TEM demonstrating size and shape of AgNPs of the prepared NSF

**Fig. 7:**
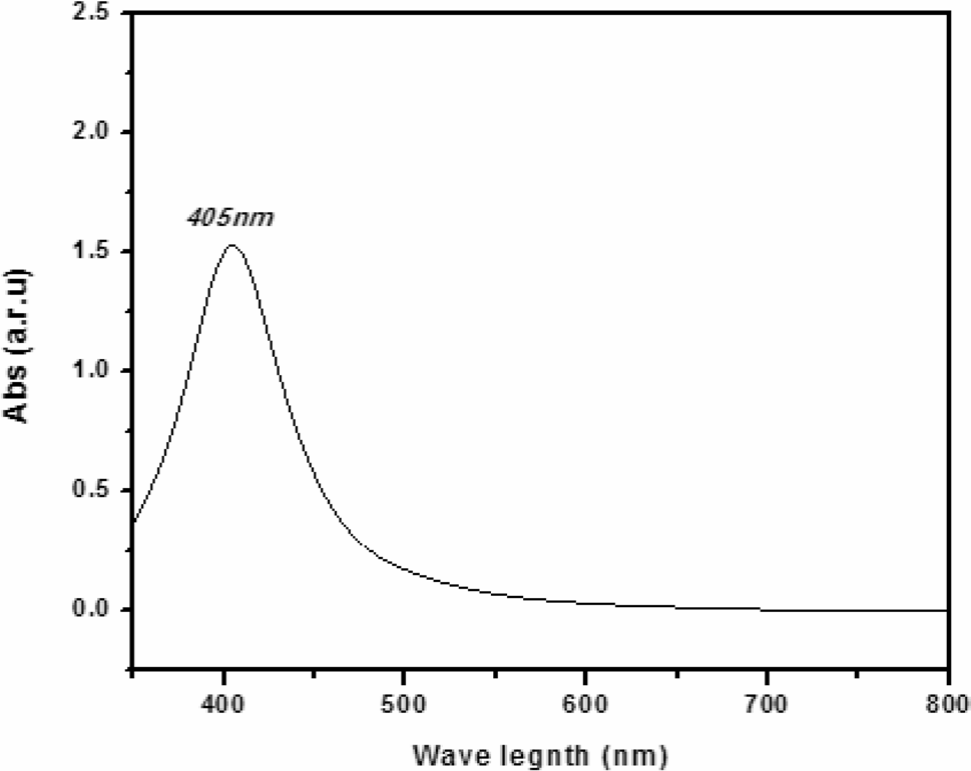
Optical Absorption of silver fluoride nanoparticles

Image analysis using TEM revealed predominantly spherical particles with an average diameter of 20 ± 5 nm (Fig. 6). UV-Vis spectrophotometry optical characterization showed a distinct absorbance peak at 405 nm, confirming the optical properties of the silver nanoparticles (Fig. 7).

### Macro-shear bond strength

Two-way ANOVA declared that dentin pretreatment and thermocycling had statistically significant effect on the shear bond strength of resin-modified glass-ionomer (*p* < 0.001) (Table 2).

**Table 2:** Comparisons and summary statistics of macro-shear bond strength (MPa) for different thermocycling conditions within each group

Group Subgroup	Macro-shear bond strength (MPa) (Mean ± SD)			*p*-value	PES (95% CI)
	Control	SDF	NSF		
*Non-aged*	7.27 ± 0.42^B, a^	7.79 ± 0.77^AB, a^	8.55 ± 0.86^A, a^	< 0.001*	0.29 (0.09 to 0.43)
*Aged*	4.22 ± 0.39^B, b^	6.11 ± 0.63^A, b^	5.96 ± 0.49^A, b^	< 0.001*	0.52 (0.32 to 0.63)
*p*-value	< 0.001*	< 0.001*	< 0.001*		
PES (95% CI)	0.70 (0.55 to 0.77)	0.41 (0.22 to 0.55)	0.62 (0.46 to 0.72)		

*PES* Partial Eta Squared, *CI* Confidence interval

Values with different superscript uppercase letters within the same horizontal row are significantly different

Values with different superscript lowercase letters within the same vertical column are significantly different

* significant (*p* < 0.05)

#### Effect of different dentin pretreatments

##### Non-aged samples (subgroup 1)

Post hoc pairwise comparisons showed that there was a significant difference between NSF and control groups (p < 0.001) and the effect size was large, 0.29. However, SDF showed nonsignificant difference when compared to both NSF and control groups. The highest bond strength was found in NSF (8.55 ± 0.86 MPa), followed by SDF (7.79 ± 0.77 MPa), while the lowest bond strength was found in the control group (7.27 ± 0.42 MPa).

##### Aged samples (subgroup 2)

Post hoc pairwise comparisons showed that the control group had significantly lower bond strength than SDF and NSF groups (p < 0.001) and the effect size was large, 0.52. Nevertheless, the difference between SDF and NSF was statistically nonsignificant. The highest bond strength was found in SDF (6.11 ± 0.63 MPa), followed by NSF (5.96 ± 0.49 MPa), while the lowest bond strength was found in the control group (4.22 ± 0.39 MPa).

#### Effect of thermocycling

Thermocycling had statistically significant impact on shear bond strength across all groups (*p* < 0.001), the non-aged samples had higher values as opposed to aged samples.

### Color change

**Fig. 8:**
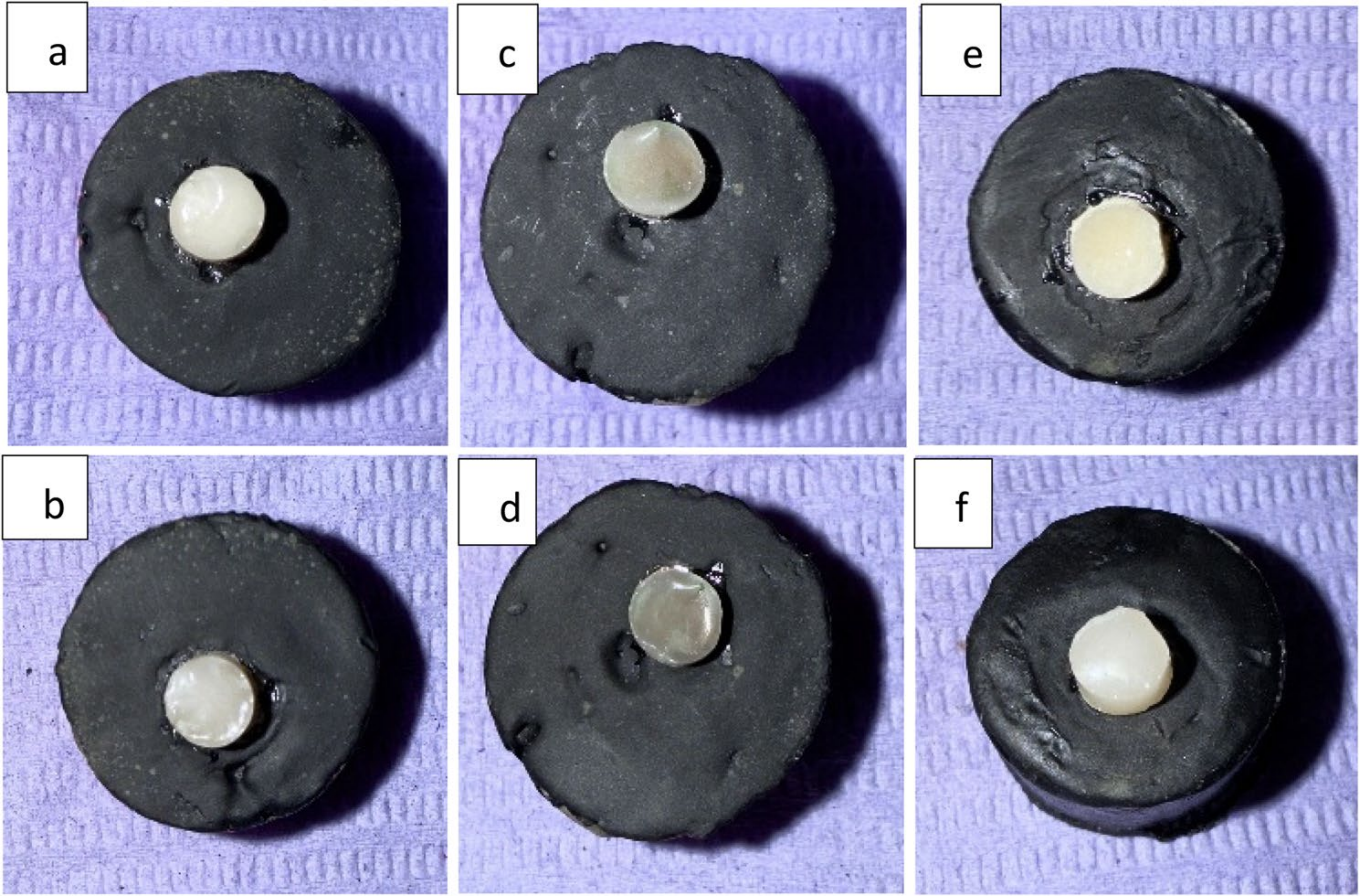
Color pre and post-thermocycling **a**- Control group before thermocycling **b**- Control after thermocycling **c**- SDF before thermocycling **d**- SDF after thermocycling **e**- NSF before thermocycling **f**- NSF after thermocycling

Post hoc pairwise comparisons showed that SDF had a significantly higher value than the other groups (*p* = 0.002) and the effect size was large, 0.45. While the difference between control and NSF groups was statistically nonsignificant. The highest value of color change (ΔE₀₀) was found in SDF (5.03 ± 1.03), followed by the control group (3.34 ± 1.09), while the lowest value was found in NSF (3.28 ± 0.75) (Table 3) (Fig. 8).

**Table 3:** Comparisons and summary statistics of color change (ΔE_00_) for different groups

(Mean ± SD)			*p*-value	PES (95% CI)
Control	SDF	NSF		
3.34 ± 1.09^B^	5.03 ± 1.03^A^	3.28 ± 0.75^B^	0.002*	0.45 (0.14 to 0.59)

*PES* Partial Eta Squared, *CI* Confidence interval

Values with different uppercase superscript letters are significantly different

* significant (*p* < 0.05)

## Discussion

In order to ensure standardized dentin demineralization across specimens, this study adopted an artificially induced caries-affected dentin (ACAD) technique, which provides uniform bonding surfaces and controlled lesion depth, reducing variability compared to natural caries-affected dentin (NCAD) [19]. Qualitative assessment was performed using polarized light microscopy (PLM), a sensitive histological technique for analyzing caries progression [18, 20]. For morphological evaluation, scanning electron microscopy (SEM) was employed, while elemental composition and mineral changes were examined through complementary energy-dispersive X-ray spectroscopy (EDX) [21].

The evaluation of bonding efficacy was performed using macro-shear bond strength testing, which is regarded as the most widely adopted method due to its simplicity, reliability and minimal technique sensitivity. Although, the micro-shear testing is better recommended [22], it was not feasible in this study because color assessment using VITA Easyshade spectrophotometer required surface areas larger than 1 mm^2^ specimens needed for micro-shear testing.

All specimens of subgroup 2 across groups were subjected to thermocycling protocols employing 5000 cycles between 5 °C and 55 °C, which are regarded suitable for simulating intraoral temperature fluctuations and replicating six months of intraoral functioning [10].

According to the findings of the present study we failed to reject the null hypothesis. Although NSF group demonstrated higher SBS than SDF in non-aged subgroups, and SDF had higher values than NSF in aged subgroups, the difference was deemed insignificant in both subgroups. However, NSF group exhibited higher as well as statistically significant difference than the control in both subgroups. The SBS of SDF group was significantly higher than that of control group regarding aged subgroups, but not high enough to be considered statistically significant in non-aged subgroups. The positively charged silver ions and silver precipitates formed following the application of SDF and NSF on dentin may enhance chemical bonding with the negatively charged carboxyl groups of RMGIC [23]. Additionally, SDF and probably NSF are capable of occluding dentinal tubules by uptake of silver ions, thereby promoting micromechanical retention of the resin component within RMGIC [24].

In the present study, the effect of SDF application on the bond strength of RMGIC was largely consistent with most published evidence, as several investigations and a recent systematic review reported no significant influence of SDF on bonding performance [8, 24–27]. Conversely, Abdelshafi et al. (2025) [28] and Shetty et al. (2022) [29] found a reduction in bond strength after SDF application, a discrepancy that could be explained by differences in testing methods, application protocols or sample sizes.

In line with the present study, findings from Das et al. (2024) [4] supported the beneficial role of NSF pretreatment, which significantly improved RMGIC bonding to carious dentin by enhancing substrate integrity, reducing dentinal fluid flow and limiting collagen degradation. Additionally, Eltoukhy et al. (2022) [30] reported a nonsignificant increase in SBS of orthodontic brackets to enamel after NSF application, highlighting that variations in substrate and bonding systems may influence outcomes.

The results obtained in this study demonstrated that thermocycling had detrimental impact on shear bond strength of RMGI in all study groups. Therefore, the null hypothesis must be rejected since the difference was statistically significant. These findings align with Ballal et al. (2019) [31], who found that thermocycling significantly reduced the SBS of GIC to dentin, likely due to interfacial degradation. The effect of thermocycling could be contributed to alterations at RMGI-dentin interface caused by thermal stresses from mismatch in coefficient of thermal expansion between dentin and RMGI, water uptake and hydrolytic degradation [31]. In contrast, they differ from Sulimany et al. (2024) [10] who observed no significant differences in SBS between immediate and delayed subgroups of RMGI, possibly due to disparity in study design, restorative material composition and conditioning protocols.

Numerous studies have addressed the discoloration effect of SDF [32–34]. However, research exploring the impact of NSF on dental tissues remains limited. To the best of current knowledge, the present study is among the pioneers to investigate and compare the discoloration effects of SDF and NSF solutions on RMGI.

The overall color change (ΔE_00_) of RMGIC-dentin complex after thermocycling was higher in SDF group (5.03 ± 1.03) in comparison to both control (3.34 ± 1.09) and NSF (3.28 ± 0.75), and the difference was regarded as statistically significant. Accordingly, the null hypothesis was totally rejected. Although the control group exhibited higher ΔE_00_ values than NSF group, it was found to be statistically insignificant. Unlike SDF, the interaction of nanosilver particles with dental tissues does not lead to the formation of oxides. Consequently, NSF is generally not associated with tooth staining, although minimal discoloration has been reported when the nanoparticle size approaches approximately 100 nm [2]. According to Paravina et al. (2019) [35], a moderately unacceptable mismatch (type a) falls between 1.8 and 3.6 ΔE_00_ units, a clearly unacceptable mismatch (type b) is between 3.6 and 5.4 units, and an extremely unacceptable mismatch (type c) is any difference above 5.4 ΔE_00_ units. Accordingly, SDF group’s ΔE_00_ values fall into the clearly unacceptable mismatch category. Whereas both control and NSF groups were in the moderately unacceptable mismatch range.

The findings of the present study are partially consistent with Karaduran et al. (2024) [2], who reported that SDF caused the greatest discoloration in carious primary molars, while NSF produced less but statistically insignificant changes compared with the control. In alignment with our findings, Espíndola-Castro et al. (2020) [36] demonstrated that NSF induced less staining than SDF. Similarly, Sayed et al. (2020) [37] found that SDF produced significantly greater staining than AgNPs, while Zhao et al. (2020) [16] confirmed the minimal discoloration associated with PEG-stabilized AgNPs compared with the pronounced color changes caused by SDF. These results, along with those of Aldosari et al. (2022) [38], who observed significant discoloration of RMGIC following SDF treatment, underscore the well-documented aesthetic drawback of SDF.

The in vitro design of this study inherently limits its ability to reproduce the complex conditions of the oral cavity and as a result, absolute control over all experimental variables cannot be attained. The exclusive use of RMGI represents another limitation of the present study. Future investigations should consider comparing the effects of SDF and NSF on staining when used with both non-light-curing restorative materials and light-curing counterparts, in order to better elucidate the influence of the light-curing process. Additionally, color changes were assessed only after placement of the filling material; therefore, the potential masking effect of the staining by restoration may have influenced the results, limiting the accurate evaluation of the true discoloration caused by the solutions. Future studies should assess color before and after placement of the restorative material to enable a more accurate comparison.

## Conclusions

Based on the current findings and within the limitations of this in-vitro study, NSF pretreatment exhibited comparable effect to SDF on the shear bond strength of RMGI bonded to artificially induced caries-affected dentin. The application of NSF prior to RMGI dramatically decreased the overall color variation of restoration-dentin complex following thermocycling as opposed to SDF. These findings advocate that NSF may be suggested as a promising alternative to SDF, emphasizing the necessity for additional research under optimized clinical settings.

## Data Availability

The datasets used and analyzed during the current study available from the corresponding author on reasonable request. All data analyzed throughout this study are presented in this published article in the form of tables and figures.

## Abbreviations

MID: Minimally invasive dentistry
SDF: Silver diamine fluoride
SMART: Silver-modified atraumatic restorative technique
GIC: Glass-ionomer cement
RMGIC: Resin-modified glass-ionomer cement
AgNPs: Silver nanoparticles
NSF: Nanosilver Fluoride
SBS: Shear bond strength
TEM: Transmission electron microscopy
SEM-EDX: Scanning electron microscopy and energy dispersive x-ray spectroscopy
PLM: Polarized light microscopy
CAD: Caries-affected dentin
PEG-AgNPs: Polyethylene glycol-coated silver nanoparticles
